# Ovarian Dropsy in a Cow

**Published:** 1896-10

**Authors:** 


Ovarian Dropsy in a Cow. Leimer, in the *Wochenschrift für Tierheilkunde*, after an examination, made a diagnosis of ovarian dropsy in a cow. When an opening was made in the region of the lower wall 120 litres of water flowed off in three hours. The cow calved the next day and remained in a healthy condition.—*Schweizer Archiv*, January and February, 1896.

